# Intraspecific Variation of Samara Dispersal Traits in the Endangered Tropical Tree *Hopea hainanensis* (Dipterocarpaceae)

**DOI:** 10.3389/fpls.2020.599764

**Published:** 2020-11-13

**Authors:** Yao-Bin Song, Xiao-Lu Shen-Tu, Ming Dong

**Affiliations:** Key Laboratory of Hangzhou City for Ecosystem Protection and Restoration, College of Life and Environmental Sciences, Hangzhou Normal University, Hangzhou, China

**Keywords:** conservation, functional traits, intraspecific variations, samara traits, wind dispersal

## Abstract

Propagule dispersal is a crucial life history stage, which affects population recruitment and regeneration as well as community structure and functions. The windborne process of samara dispersal is affected not only by samara traits and other plant traits, but also by environmental factors. Therefore, studying samara traits related to its dispersal and intraspecific variation in relation to other plant traits and environmental factors could help to understand population distribution and dynamics. *Hopea hainanensis*, a Dipterocarpaceae tree species dominant in lowland rainforests in Hainan (China) but endangered due to anthropogenic disturbances, is dispersed mainly by wind because of its sepal-winged samara. Here, we measured dispersal-related intraspecific samara traits of *H. hainanensi*s, and analyzed their variation and correlation in relation to plant height, DBH (diameter at breast height), and elevation plant location. Great variations in the samara traits existed, and the variations were larger within than among individuals, which indicated a “bet-hedging” strategy of this species. Plant height, DBH, and elevation explained slight variation in the samara traits. Samara dispersal potential is mainly affected by the samara mass and morphological traits. Samara settling velocity was significantly positively correlated with fruit mass, seed mass, length and width, as well as samara wing loading, and negatively correlated with wing mass ratio, wing area, and wing aspect ratio. Substantial proportions of intraspecific variation in samara dispersal are explained by the samara mass and morphological traits. Natural regeneration with human-aided dispersal is necessary for recovering the *H. hainanensis* population. This finding contributes to the generalization of trait-based plant ecology, modeling of seed dispersal in tropical forests, and conservation and recovery of rare and endangered species such as *H. hainanensis*.

## Introduction

Plant functional traits are morphological, physiological, and phenological characteristics by which plants interact with their environment during evolutionary processes. Such traits link ecological processes on multiple scales, from individual, population, community, and ecosystem to landscape (McIntyre et al., 1999; Díaz et al., 2004; Suding et al., 2008; Pérez-Harguindeguy et al., 2013). Trait-based approaches in plant ecology have provoked significant progress in population demography (Struckman et al., 2019), species distribution models (Benito Garzón et al., 2019), community assembly (Ackerly and Cornwell, 2007), ecosystem function (Cornwell et al., 2008), global vegetation models (van Bodegom et al., 2014), prediction of ecosystem responses to global changes (Diamond et al., 2012), and evaluation of ecosystem services (Díaz et al., 2007, 2011), which has been a new paradigm in ecology (Wright et al., 2004; van Bodegom et al., 2014; Díaz et al., 2016; He et al., 2019, 2020).

Rare and endangered plant species usually have populations composed of few individuals restricted to local communities and ecosystems. Based on the mass ratio hypothesis (Grime, 1998), community and ecosystem processes and functions are mainly determined by dominant (or sub-dominant, or both) species, instead of endangered species and their traits (Díaz et al., 2007; Lohbeck et al., 2015). While, rare species are also playing important roles in ecosystem functioning, especially for those species with unique traits and their intraspecific variations (Lyons and Schwartz, 2001; Mouillot et al., 2013; Jain et al., 2014), and their propagule dispersal is critical for the maintenance of biodiversity. Identifying and measuring functional traits related to responses to environmental changes and ecosystem function of endangered species could contribute to understanding the mechanism of being endangered or threatened and its implications for conservation practice (Chown, 2012; Cochrane et al., 2015; Turner et al., 2017; Álvarez-Yépiz et al., 2019), especially under the uncertainty of climate changes in the future (Di Musciano et al., 2020).

Propagule (e.g., seed) dispersal is one of the most important stages in plant life history (Harper, 1977; Bonte and Dahirel, 2017). As sessile organisms, plants increase population size and distribution and cope with environmental stochasticity and uncertainty through propagule dispersal (Cochrane et al., 2015; Beckman et al., 2020). Seed dispersal traits (and their variations), such as seed size, mass, and dispersal mode affect dispersal distance and potential, seedling emergence and survival, plant colonization and growth (Janzen, 1970; Saatkamp et al., 2019; Schupp et al., 2019; Snell et al., 2019; Beckman et al., 2020). These dispersal traits impact population dynamics, interspecific interactions, population regeneration, community assembly and succession, and ecosystem service delivery (Grubb, 1977; Ribeiro et al., 2016; Saatkamp et al., 2019). Many studies have focused on soft traits (i.e., easily measured traits) related to seed dispersal at the interspecific level (e.g., Tamme et al., 2014; Thomson et al., 2018); however, some studies found that intraspecific variation (including within species and within-individual) of seed dispersal traits may be considerable (Wyse et al., 2019), but this has not been explored (Snell et al., 2019; Wyse et al., 2019; Chen and Giladi, 2020).

Samara (i.e., winged seed) is present in 25 orders, 45 families, and 140 genera of angiosperms (der Weduwen and Ruxton, 2019). It contributes to long-distance dispersal of seeds by wind (Augspurger and Franson, 1987; Greene and Johnson, 1990; Nathan et al., 2002). Dipterocarpaceae has 16 genera and approximately 500 species widely distributed in Asian tropical forests. Seeds from this family usually are bract-winged samaras possessing aerodynamic behavior of helicopters characterized by relatively stable flight, explicit dispersal direction, and long dispersal distance (Augspurger, 1986). Dispersion-related traits of Dipterocarpaceae samaras display substantial inter and intraspecies variations (Sipe and Linnerooth, 1995); however, such traits and their potential have not been fully described (der Weduwen and Ruxton, 2019).

Due to evolutionary adaptation, seed trait tradeoffs (Saatkamp et al., 2019), such as seed size vs. seed production and dispersal ability vs. colonization ability (Moles and Westoby, 2006), are common among and within species. Exploring the tradeoff among different seed traits (especially for intraspecies) is vital to elucidate the potential evolution ability of endangered species and their population dispersal (Huang et al., 2016; Saatkamp et al., 2019).

*Hopea hainanensis* Merr. *et* Chun, is a Dipterocarpaceae species distributed in the northern tropic (Hainan in China and Nghe An in Vietnam). Its samaras (seeds) dispersed mainly by wind due to its sepal-winged samaras. It was once a dominant species in lowland tropical rainforests; nevertheless, it is currently listed as an endangered species in IUCN (Ly et al., 2018) and first-class state protection wild plants in China, mainly due to anthropogenic disturbances, such as commercial logging and shifting cultivation (Guo and Zang, 2013; Lu et al., 2020). Current ecological and conservation biology for *H. hainanensis* mainly focus on seed germination (Wen et al., 2002), habitat characteristics, seedling banks (Pei et al., 2015; Lu et al., 2020), and population structure (Fu et al., 2019; Zhang et al., 2019). Although little information has been found so far about the functional traits of Dipterocarpaceae species producing winged seeds, especially for samara traits related to its population regeneration and maintenance, the information would help to predict their population dynamics and development trends and to understand their adaptive strategies. In this study, we sampled and measured samaras of *H. hainanensis* from natural populations in a tropical mountain cloud forest, located in Bawangling Nature Reserve, Hainan Island, South China, to answer the following scientific questions: (1) What are intraspecific variations of samara traits? (2) Are such traits related to intrinsic or extrinsic factors? (3) Is there any tradeoff among samara traits in *H. hainanensis*? and (4) Which samara traits affect samara settling velocity in *H. hainanensis*?

## Materials and Methods

### The Species

*Hopea hainanensis* is an evergreen tree, *ca*. 25 m in height, naturally distributed in valley and windless lower foothills at 300–900 m a.s.l. in lowland rainforests. A samara of *H. hainanensis* is composed of an ovoid main body (seed covered by pericarp hereinafter referred to as seed) and two sepal-wings oppositely attached to the seed (Tan et al., 2018). The thousand-kernel weight of *H. hainanensis* without any wings or appendages is *ca*. 300 g (Institute of Guangdong Forestry Science, 1964). The seeds of *H. hainanensis* are typically recalcitrant with higher moisture content, short life span, and intolerance to dehydration and storage (Wen et al., 2002). Additionally, population regeneration of *H. hainanensis* might be affected by negative density dependence, e.g., seedlings aggregately distributed 0–5 m from the mother trees (Lu et al., 2020), which suffered over 65% herbivory (Pei et al., 2015). In the previous example, there was a substantial barrier hindering seedling growth to saplings of *H. hainanensis* (Lu et al., 2020).

Bawangling Nature Reserve, Hainan Island, South China (108°58′–109°53′ E, 18°53–19°20′ N) is the main distribution region of *H. hainanensis* in China. The climate is tropical monsoon, with mean annual temperature and precipitation of 24.2°C and 1677.1 mm, respectively. Zonal vegetation is lowland rainforests, mountain rainforests, and mountain evergreen and dwarf forests (Long et al., 2015).

### Field Sampling

We sampled ripe samaras from 17 mature, healthy, and high seed-setting rate *H. hainanensis* trees from natural populations in Bawangling Nature Reserve. A total of 3,207 samaras were collected, including 876 samaras (51.5 ± 3.9 samaras per trees in average) with intact wings randomly collected from each tree. For the 17 trees, we also measured DBH (diameter at breast height, i.e., 1.5 m above the ground; 23–64 cm), height (13–33 m), and elevation of locality (270–840 m. a.s.l.). All fresh samples were carefully numbered and transported to the laboratory as soon as possible for further measurements.

### Measurement

Intact samaras were weighed to determine fruit mass (*F*_mass_, g), then each intact samara was used to determine samara settling velocity (SSV, m s^–1^, Andersen, 1992) in still air, shortly after field sampling. Each samara was released from the top of a 21.2 m-high building in still air, and the falling time was recorded by two independent persons. Each samara was measured 3 times; the measurement was averaged for the falling time of each samara. To keep the samara intact, a soft sponge cushion was laid on the ground. SSV was calculated by the releasing height divided by the falling time (Andersen, 1992), i.e., lower settling velocity means longer time of dispersal (indicating longer distance of dispersal). This measurement assumes that samara attains terminal velocity instantaneously (Andersen, 1992), but it is not precisely equivalent to the terminal velocity (Augspurger, 1986).

After measurements of SSV, samara wings and seeds (i.e., wing-removed samara) were carefully separated and weighed to determine wing mass (*W*_mass_, g) and seed mass (*S*_mass_, g), respectively. Seed length (SL, cm) and width (SW, cm) were measured using a Vernier caliper. Detached-wing length (WL, cm), width (WW, cm), and area (WA, cm^2^) were assessed using the WinFOLIA Leaf Analysis Software (Regent Instruments, Quebec City, QC, Canada). To reduce variations in weight and morphology caused by seed desiccation, all measurements were completed within 1 week after collection.

### Data Analysis

Wing mass ratio (WMR) was determined using *W*_mass_/*F*_mass_. Seed morphological index (SMI) was calculated as SL/SW. Wing aspect ratio was calculated using WL/WW. Wing loading (WL) was calculated by dividing the samara’s mass by its wing area (Wyse et al., 2019). To explore samara variations within species and individual mother plants, all the traits were subjected to Kruskal–Wallis test with 999 times permutation test. To explore the interrelationships among samara traits, a Spearman correlation analysis was conducted. A generalized linear model (GLM) was used to examine the relationships between samara traits and plant height, plant DBH and elevation, and the relationships between samara traits and SSV of *H. hainanensis*. All analyses were performed in R 4.0.2 (R Core Team, 2020).

## Results

### Decomposition of Samara Trait Variations

**FIGURE 1:**
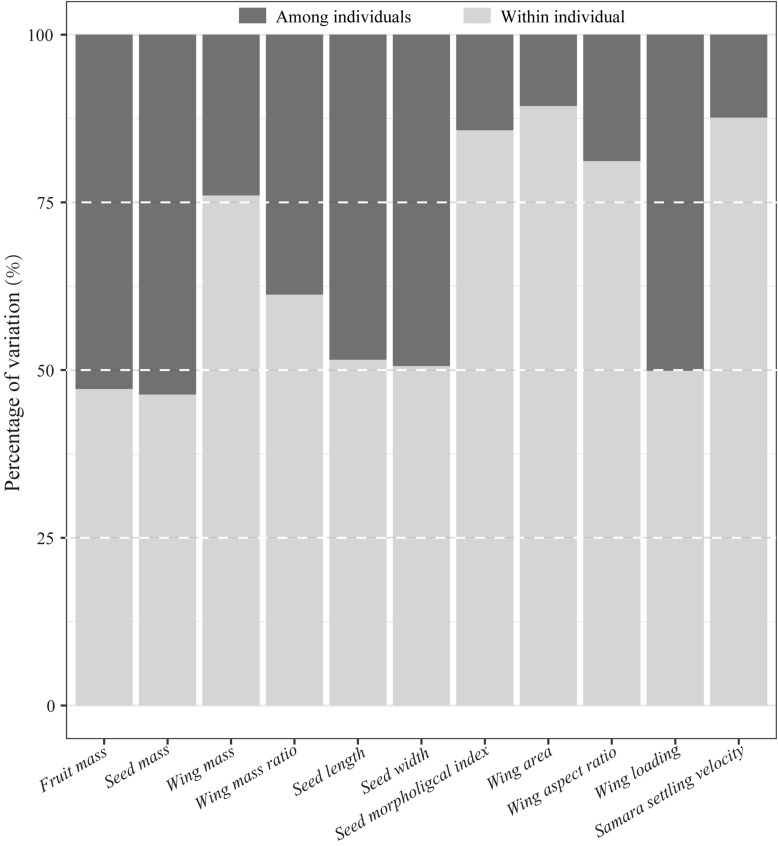
Variation decomposition of samara traits of *Hopea hainanensis* within- and among individuals.

Samara traits of *H. hainanensis* showed substantial intraspecific variations, both among and within individuals (Table 1 and Figure 1). Mass-related traits (i.e., fruit, seed, and wing mass, and WMR) showed large variations (>22%), while morphological traits of seeds (length, width, and SMI) had relatively small variations (<9%). The wing area had a larger variation than the wing aspect ratio among different samaras (20.34 and 14.84%, respectively). The largest variations detected were in wing loading and SSV (28.57 and 35.65%, respectively). The SSV ranged from 1.39 to 9.02 m s^–1^ among different samaras.

**TABLE 1:** Distribution characteristics of samara traits and Kruskal–Wallis test (*χ*^2^) among trees of *Hopea hainanensis*.

Samara trait	Mean	Median	Minimum	Maximum	SD	CV (%)	*χ*^2^
*F*_mass_ (g)	0.87	0.87	0.30	1.43	0.21	24.14	469.77**
*S*_mass_ (g)	0.69	0.69	0.13	1.25	0.18	26.09	484.32**
*W*_mass_ (g)	0.18	0.18	0.08	0.30	0.04	22.22	217.33**
*WMR* (g g^–1^)	0.21	0.21	0.09	0.56	0.05	23.81	359.56**
*SL* (cm)	1.51	1.53	1.02	1.85	1.21	7.99	426.66**
*SW* (cm)	1.06	1.06	0.70	1.31	0.92	8.70	419.35**
*SMI* (cm cm^–1^)	1.43	1.43	1.06	1.72	0.08	5.59	130.18**
*WA* (cm^2^)	12.83	12.96	4.63	21.61	2.61	20.34	91.49**
*WAR* (cm cm^–1^)	3.64	3.62	1.98	5.48	0.54	14.84	181.40**
*WL* (g cm^–2^)	0.07	0.07	0.02	0.20	0.02	28.57	434.49**
*SSV* (m s^–1^)	4.74	4.53	1.39	9.02	1.69	35.65	103.28**

F_mass_, S_mass_, W_mass_, WMR, SL, SW, SMI, WA, WAR, WL, and SSV stand for fruit (i.e., intact samara) mass, seed (i.e., wing-removed samara) mass, wing mass, wing mass ratio, seed length, seed width, seed morphological index, wing area, wing aspect ratio, wing loading, and samara settling velocity, respectively; **P < 0.01.

Results of the Kruskal–Wallis test showed significant differences among trees for all samara traits (Table 1). Over 50% of the variations in almost all samara traits were explained by variation within individual mother trees (Figure 1). Moreover, variations among individuals for wing mass, wing area, wing aspect ratio, SMI, and SSV explained less than 25% of the total variations (Figure 1).

### Relationships Between Samara Traits and Intrinsic and Extrinsic Factors

**FIGURE 2:**
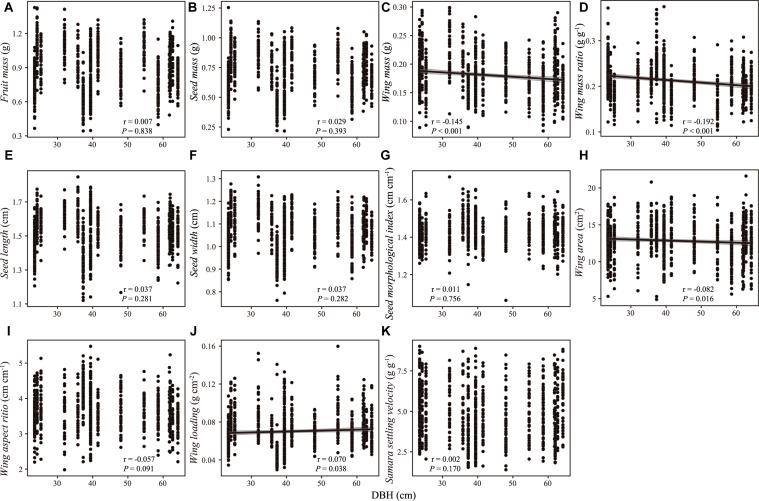
Correlation between samara traits [**(A)** fruit mass; **(B)** seed mass; **(C)** wing mass; **(D)** wing mass ratio; **(E)** seed length; **(F)** seed width; **(G)** seed morphological index; **(H)** wing area; **(I)** wing aspect ratio; **(J)** wing loading; **(K)** samara settling velocity] and DBH of *Hopea hainanensis*. Regression lines with 95% interval (shaded area) were plotted for significant relationships with *P* < 0.05.

**FIGURE 3:**
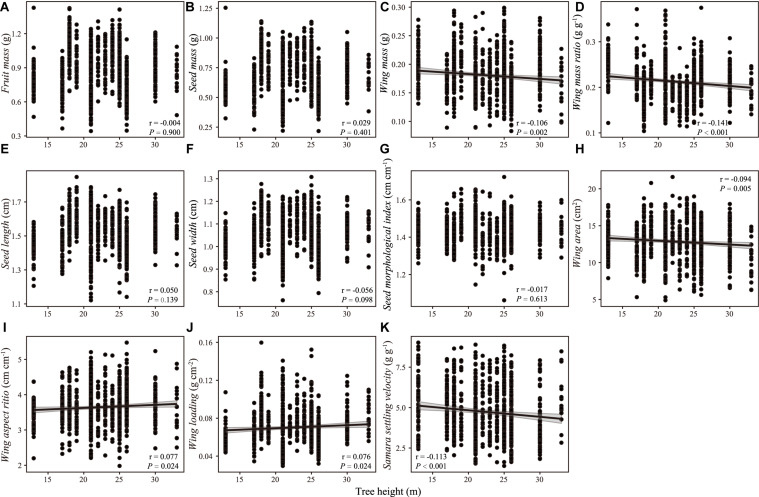
Correlation between samara traits [**(A)** fruit mass; **(B)** seed mass; **(C)** wing mass; **(D)** wing mass ratio; **(E)** seed length; **(F)** seed width; **(G)** seed morphological index; **(H)** wing area; **(I)** wing aspect ratio; **(J)** wing loading; **(K)** samara settling velocity] and tree height of *Hopea hainanensis*. Regression lines with 95% interval (shaded area) were plotted for significant relationships with *P* < 0.05.

**FIGURE 4:**
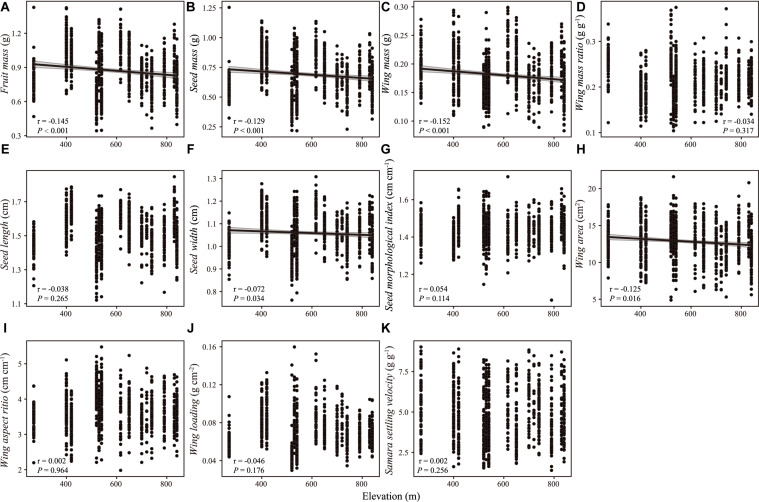
Correlation between samara traits [**(A)** fruit mass; **(B)** seed mass; **(C)** wing mass; **(D)** wing mass ratio; **(E)** seed length; **(F)** seed width; **(G)** seed morphological index; **(H)** wing area; **(I)** wing aspect ratio; **(J)** wing loading; **(K)** samara settling velocity] of *Hopea hainanensis* and elevation. Regression lines with 95% interval (shaded area) were plotted for significant relationships with *P* < 0.05.

There were weak correlations between samara traits and intrinsic (i.e., DBH, tree height, Figures 2, 3) and extrinsic factors (i.e., elevation, Figure 4). Most samara traits showed no significant relationships with DBH of mother trees (Figure 2), except wing mass (*r* = –0.145, *P* < 0.001), WMR (*r* = –0.192, *P* < 0.001), and wing area (*r* = –0.082, *P* = 0.016), showing weak but significant negative correlation with DBH; and wing loading showed weak, significant positive correlation with DBH (*r* = 0.070, *P* = 0.038). The relationships between samara traits and tree height showed almost the same trends as those of samara traits and DBH, except regarding wing area ratio, which showed a weak positive correlation (*r* = 0.077, *P* = 0.024); whereas SSV showed a weak negative correlation (*r* = –0.113, *P* < 0.001) with tree height (Figure 3). Some samara traits (i.e., fruit, seed and wing mass, seed width, and wing area) decreased with the elevation a.s.l. (mother tree location), while other traits showed no significant correlation with elevation (Figure 4).

### Correlations Among Samara Traits

Significant correlations between the mass and morphological traits of samaras were present (Table 2). Fruit, seed and wing mass, SL and width, and wing area were positively correlated to each other, with the highest correlation between fruit and seed mass (*r* = 0.99, *P* < 0.01, Table 2). Wing loading was positively correlated with fruit and seed mass and SL and seed width, but negatively correlated with WMR, SMI, wing area, and aspect ratio (Table 2). However, wing mass showed no significant relationship with wing loading (*r* = –0.01, *P* = 0.798, Table 2).

**TABLE 2:** Spearman correlation coefficients among samara traits of *Hopea hainanensis*.

	*F*_mass_	*S*_mass_	*W*_mass_	*WMR*	*SL*	*SW*	*SMI*	*WA*	*WAR*
*S*_mass_	**0.99**								
*W*_mass_	**0.62**	**0.51**							
*WMR*	**−0.56**	**−0.67**	**0.24**						
*SL*	**0.84**	**0.84**	**0.52**	**−0.51**					
*SW*	**0.88**	**0.89**	**0.51**	**−0.57**	**0.80**				
*SMI*	**−0.20**	**−0.22**	**−0.08**	**0.18**	**0.13**	**−0.44**			
*WA*	**0.30**	**0.20**	**0.73**	**0.40**	**0.19**	**0.19**	**−**0.03		
*WAR*	**−0.27**	**−0.30**	**−**0.06	**0.31**	**−0.28**	**−0.29**	0.07	**0.15**	
*WL*	**0.65**	**0.72**	**−**0.01	**−0.84**	**0.60**	**0.64**	**−0.17**	**−0.48**	**−0.37**

*F_mass_, S_mass_, W_mass_, WMR, SL, SW, SMI, WA, WAR, and WL stand for fruit (i.e., intact samara) mass, seed (i.e., wing-removed samara) mass, wing mass, wing mass ratio, seed length, seed width, seed morphological index, wing area, wing aspect ratio, and wing loading, respectively. Bold values indicate statistical significance at P < 0.05.*

**FIGURE 5:**
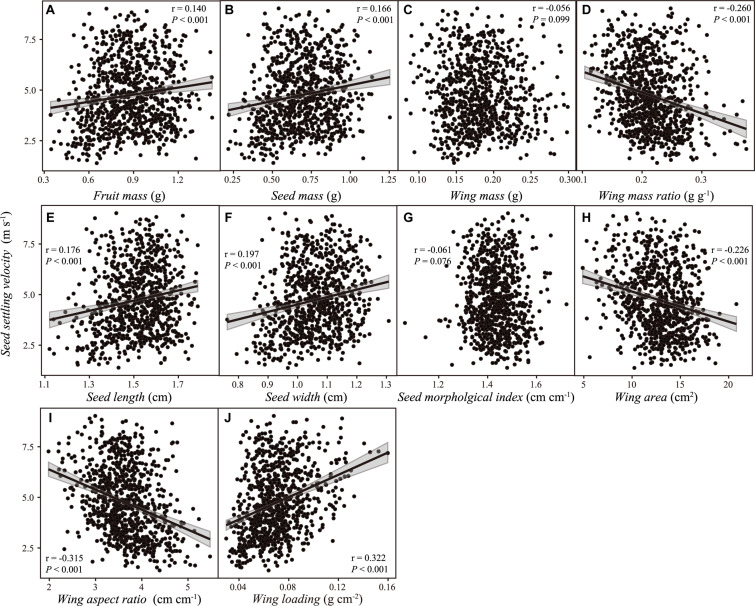
The relationship between samara traits [**(A)** fruit mass; **(B)** seed mass; **(C)** wing mass; **(D)** wing mass ratio; **(E)** seed length; **(F)** seed width; **(G)** seed morphological index; **(H)** wing area; **(I)** wing aspect ratio; **(J)** wing loading] and samara settling velocity of *Hopea hainanensis*. Regression lines with 95% interval (shaded area) were plotted for significant relationships with *P* < 0.05.

No significant relationship was detected, neither between SSV and wing mass (*r* = –0.056, *P* = 0.099), nor between SSV and SMI (*r* = –0.061, *P* = 0.076). SSV was positively correlated with fruit and seed mass, SL and seed width, and wing loading, but negatively correlated with WMR, wing area and aspect ratio (Figure 5). Those correlations were consistent even considering the potential dependence of data from the same maternal tree, except the relationship between SSV and wing area (*r* = –0.242, *P* = 0.349; Supplementary Figure 1).

## Discussion

In this study, we found substantial intraspecific variations in samara traits of *H. hainanensis*, both among and within individuals, and within-individual variation was larger than among-individual variation. Its samara dispersal potential was mainly affected by the samara mass and morphological traits. Substantial proportions of intraspecific variation in its samara dispersal were explained by the samara mass and morphological traits. However, plant height, DBH, and elevation seems independent of variation in the samara traits.

### Intraspecific Variations in Samara Traits

Intraspecific seed variations are considered to have important evolutionary and ecological implications (Sipe and Linnerooth, 1995). If among-individual variations are higher than within-individual variations, it could be inferred that heritable seed traits might be subjected to ecological and evolutionary selection in communities (Sipe and Linnerooth, 1995; Herrera, 2017; Wyse et al., 2019). Whereas higher within-individual seed variations are thought be a “bet-hedging” strategy to adapt to spatial and temporal environmental changes (Herrera, 2017), which is barely subjected to ecological and evolutionary selections. Most of the samara trait variations of *H. hainanensis*, including SSV, were found within individuals. This result implies that samaras of *H. hainanensis* might adopt a “bet-hedging” strategy (Herrera, 2017; Wyse et al., 2019) in coping with environmental heterogeneity in lowland tropical cloud forests, such as those in the Bawangling Natural Reserve (Long et al., 2015).

### Relationships Between Samara Traits and Intrinsic and Extrinsic Factors

Factors related to seed intraspecific variations can be decomposed into intrinsic (e.g., plant height and age and growth status) and extrinsic (e.g., local microclimate, soil nutrient, and elevation) factors (Schupp et al., 2019). In this study, we used tree height and DBH as surrogate indices of *H. hainanensis* age (Long et al., 2015). It is difficult to measure the actual age of tropical trees using ordinary dendrochronology protocols because of the absence of clear annual growth rings (Rozendaal and Zuidema, 2011). Interspecies comparison studies found that taller species tend to have larger seeds compared to shorter species (Díaz et al., 2016). However, we encountered limited and weak correlations between samara traits and tree age, even though we sampled a substantial range of tree heights (13–33 m) and DBH (23–64 cm) in *H. hainanensis*, which was consistent with Clark et al. (2005) and Augspurger et al. (2016, 2017); these authors reported that no tree traits could be predictive of seed traits, including dispersal distances.

Two contrasting hypotheses have been used to explain the relationship between seed traits and elevation – the “stress-tolerance” hypothesis and the “energy constraints” hypothesis (Qi et al., 2014). The “stress-tolerance” hypothesis claims that larger seeds have more advantages in coping with the stressful environments in higher elevation (Pluess et al., 2005), whereas, based on the “energy constraints” hypothesis (Qi et al., 2014), seed mass and morphological traits may be negatively correlated with elevation, as lower temperatures inhibit leaf photosynthesis and seed development at higher elevations (Qi et al., 2014, 2015). In our study, we found that samara size- related traits (i.e., mass) decreased with the increase in elevation, which seems to support the “energy constraints” hypothesis. In other words, samaras at lower elevations might have a higher advantage regarding samara mass than those at higher elevations, which would lead to higher seed germination and seedling growth rates. On the other hand, no significant relationship between samara settling velocity (at windless status) and elevation was verified. Nevertheless, the Bawangling Natural Reserve frequently suffers from strong wind disturbances (e.g., typhoons) (Yang et al., 2017), which has been found to contribute to seed dispersal of dominant species such as *Dacrydium pierrei* in this area (Wu et al., 2018). In this context, samaras at higher elevations would disperse farther due to higher wind speed and smaller seed mass, according to the estimated seed dispersal distance formula proposed by Cremer (1977). These results suggest a (weak) tradeoff between seedling establishment and seed dispersal distance (Meyer and Carlson, 2001; Fricke et al., 2019; Chen and Giladi, 2020) for *H. hainanensis*. Additionally, these results suggest that intraspecific variation in the samara traits of this wind-dispersed species cannot be explained by the intrinsic (i.e., plant height and DBH) or extrinsic (elevation) factors we explored in this study. Other factors such as genetic and edaphic factors need to be considered and might explain those intraspecific variations in future.

### Relationships Between Samara Traits and Samara Settling Velocity

Seed dispersal is a mechanism that allows plants to cope with environmental change, stochasticity, and uncertainty (Cochrane et al., 2015). Seed traits (e.g., seed mass and seed morphology) and their variations are closely related to seed behavior and dispersal distance (Sonkoly et al., 2017). Thus, seed mass and morphology are usually used to estimate seed dispersal potential (Augspurger, 1986; Minami and Azuma, 2003). Our study found that samara mass-related traits and samara morphological traits were inter-correlated with each other, and eight of those ten samara traits we studied explained variations in SSV. This result suggests that the samara dispersal potential of *H. hainanensis* is highly affected by samara traits, which may have profound implications for population demography and genetics of *H. hainanensis*.

Furthermore, we found weak but significant evidence of a positive relationship between samara seed mass and SSV. This might imply that the large seeds of *H. hainanensis* may have better seedling performance but inferior dispersal, as found in other species (Saatkamp et al., 2019). Interestingly, wing morphological traits (e.g., wing area and wing aspect ratio) and dispersal investment (i.e., wing mass ratio) – instead of wing mass – showed negative relationships with SSV. Thus, samaras with larger wing area (and with larger wing mass ratio, Table 2) can disperse farther from mother trees. In other words, *H. hainanensis* invests less biomass on wings, for dispersal. Interspecies comparisons from 83 wind-dispersed species also discovered that there was no significant relationship between maximum plant height and dispersal investment (Thomson et al., 2018). However, we do not know if samara wings have other ecological functions aside from dispersal, for example, affecting seedling emergence or growth, or both.

Wing loading is usually used as an indicator of dispersal potential (Andersen, 1993; Liang et al., 2020; Wyse and Hulme, in press). Previous studies found wing loading (or its square root) may account for 40–80% of total variation in descent rate (Matlack, 1987; Sipe and Linnerooth, 1995; Augspurger et al., 2016; Wyse et al., 2019) of some species, but few studies examined this trait in Dipterocarpaceae species with sepal-winged samaras, such as *H. hainanensis*. In our study, we also verified that samara wing loading is the most effective predictor of SSV among 10 samara morphological and size traits of *H. hainanensis*, which explains 56.7% of total variations of SSV (Figure 5).

### Conservation Implications for *H. hainanensis*

A previous study observed that the seeds of *H. hainanensis*, which are typically recalcitrant, have a higher germination rate but shorter lifespan than other Dipterocarpaceae species (Wen et al., 2002). This suggests that samara dispersal would play an important role on seed fate and determine whether the seed can arrive to a “safe site” to germinate. A recent study found that the samaras of *H. hainanensis* failed to spread from the mother trees (Lu et al., 2020). Actually, the dense understory could have prevented the dispersal by changing the understory aerodynamics or samaras could have intercepted by the understory plants, even if the samaras could potentially be wind-dispersed further away. This implies that human-aided natural regeneration is necessary for recovering the *H. hainanensis* populations. Different approaches could be used to this end, such as removing part of the litters and understory to improve the understory’s aerodynamics, helping samaras to disperse and decreasing the chance of samaras aggregating with each other. In another moment, transport some fallen samaras that aggregated around mother trees and are yet to germinate to new “safe sites” (e.g., flat landform with slightly acidic soil and higher soil phosphorus content) (Lu et al., 2020). Another option would be to transplant some fresh seedlings that are aggregating around mother trees to other sites.

### Limitations

In this study, we only focused on the main distribution area of *H. hainanensis*. The findings in our study might be applied to other populations only with caution due to potential population genetic divergence. As such, future studies on samara trait variations of *H. hainanensis* among multiple populations are needed. Another limitation is that we only studied SSV in still air; the flight behavior and dispersal distance of *H. hainanensis* samaras would be more complicated in windy environments. Thus, field investigation of the spatial distribution pattern of seed rain and seed germination characteristics and wind-tunnel experiments (e.g., Liang et al., 2020) of *H. hainanensis* would provide more information on samara dispersal potential.

## Conclusion

Substantial intraspecific (both among and within individuals) variations of samara traits in *H. hainanensis* were found. The within-individual variation was higher than the among-individual one, which indicates a “bet-hedging” strategy of *H. hainanensis*. Intrinsic (plant height and DBH) and extrinsic (elevation) factors could explain little regarding variations in samara traits. We verified that the samara dispersal potential of *H. hainanensis* was mainly affected by its mass and morphological traits.

## Data Availability Statement

The raw data supporting the conclusions of this article will be made available by the authors, without undue reservation.

## Author Contributions

Y-BS and MD contributed to the study conception and design. Y-BS and X-LS-T collected the data. All authors wrote and reviewed the manuscript and have read and approved the final manuscript.

## Conflict of Interest

The authors declare that the research was conducted in the absence of any commercial or financial relationships that could be construed as a potential conflict of interest.

## Supplementary Material

Supplementary Figure 1 The relationship between samara traits [**(A)** fruit mass; **(B)** seed mass; **(C)** wing mass; **(D)** wing mass ratio; **(E)** seed length; **(F)** seed width; **(G)** seed morphological index; **(H)** wing area; **(I)** wing aspect ratio; **(J)** wing loading] and samara settling velocity of *Hopea hainanensis*. Each point represents the mean trait value of one parent tree with bars representing one standard error around the mean (*n* = 17 parent trees). Regression lines with 95% interval (shaded area) were plotted for significant relationships with *P* < 0.05.
