# Supplementary material for: Intraspecific Variation of Samara Dispersal Traits in the Endangered Tropical Tree Hopea hainanensis (Dipterocarpaceae)
Source: Front Plant Sci. 2020 Nov 13;11:599764. doi: 10.3389/fpls.2020.599764 (PMC7691252; doi:10.3389/fpls.2020.599764)

Supplementary Material

Intraspecific variation of samara dispersal traits in the endangered tropical tree *Hopea hainanensis* (Dipterocarpaceae)

Yao-Bin Song[1], Xiao-Lu Shen-Tu[1], Ming Dong[1*]

***Correspondence:**
Ming Dong: dongming@hznu.edu.cn

FIGURE 1 The relationship between samara traits (A, fruit mass; B, seed mass; C, wing mass; D, wing mass ratio; E, seed length; F, seed width; G, seed morphological index; H, wing area; I, wing aspect ratio; J, wind loading) and samara settling velocity of *Hopea hainanensis*. Each point represents the mean trait value of one parent tree with bars representing one standard error around the mean (n = 17 parent trees). Regression lines with 95% interval (shaded area) were plotted for significant relationships with P < 0.05.

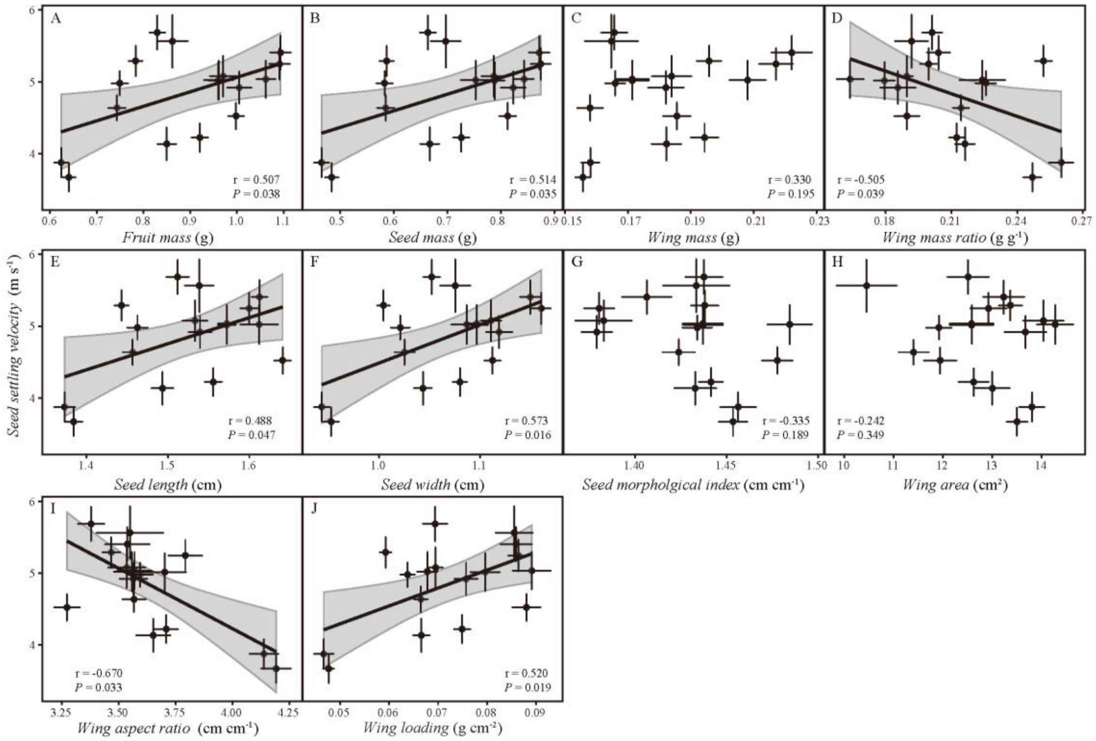

Supplement: Supplementary Figure 1 — The relationship between samara traits [(A) fruit mass; (B) seed mass; (C) wing mass; (D) wing mass ratio; (E) seed length; (F) seed width; (G) seed morphological index; (H) wing area; (I) wing aspect ratio; (J) wing loading] and samara settling velocity of Hopea hainanensis. Each point represents the mean trait value of one parent tree with bars representing one standard error around the mean (n = 17 parent trees). Regression lines with 95% interval (shaded area) were plotted for significant relationships with P < 0.05. [file Image_1.pdf]
